# Genome-wide mapping of GlnR-binding sites reveals the global regulatory role of GlnR in controlling the metabolism of nitrogen and carbon in *Paenibacillus polymyxa* WLY78

**DOI:** 10.1186/s12864-023-09147-1

**Published:** 2023-02-23

**Authors:** Tianshu Wang, Xiyun Zhao, Xinyuan Wu, Sanfeng Chen

**Affiliations:** 1 State Key Laboratory for Agrobiotechnology, College of Biological Sciences and Key Laboratory of Soil Microbiology of Agriculture Ministry, China Agricultural University, Beijing, People’s Republic of China; 2 Institute of Agricultural Resources and Regional Planning, Chinese Academy of Agricultural Sciences, Beijing, People’s Republic of China

**Keywords:** *Paenibacillus polymyxa*, GlnR, GlnR-binding site, Transcriptional regulator, Nitrogen fixation

## Abstract

**Background:**

*Paenibacillus polymyxa* WLY78 is a Gram-positive, endospore-forming and N_2_-fixing bacterium. Our previous study has demonstrated that GlnR acts as both an activator and a repressor to regulate the transcription of the *nif* (*ni*trogen *f*ixation) operon (*nifBHDKENXhesAnifV*) according to nitrogen availability, which is achieved by binding to the two GlnR-binding sites located in the *nif* promoter region. However, further study on the GlnR-mediated global regulation in this bacterium is still needed.

**Results:**

In this study, global identification of the genes directly under GlnR control is determined by using chromatin immunoprecipitation-quantitative PCR (ChIP-qPCR) and electrophoretic mobility shift assays (EMSA). Our results reveal that GlnR directly regulates the transcription of 17 genes/operons, including a *nif* operon, 14 nitrogen metabolism genes/operons (*glnRA*, *amtBglnK*, *glnA1*, *glnK1*, *glnQHMP*, *nasA*, *nasD1*, *nasD2EF*, *gcvH*, *ansZ*, *pucR*, *oppABC*, *appABCDF* and *dppABC)* and 2 carbon metabolism genes (*ldh3* and *maeA1*). Except for the *glnRA* and *nif* operon, the other 15 genes/operons are newly identified targets of GlnR. Furthermore, genome-wide transcription analyses reveal that GlnR not only directly regulates the expression of these 17 genes/operons, but also indirectly controls the expression of some other genes/operons involved in nitrogen fixation and the metabolisms of nitrogen and carbon.

**Conclusion:**

This study provides a GlnR-mediated regulation network of nitrogen fixation and the metabolisms of nitrogen and carbon.

**Supplementary Information:**

The online version contains supplementary material available at 10.1186/s12864-023-09147-1.

## Background

Nitrogen is one of the essential elements of life and an important constituent of various cellular metabolites and macromolecules. To all bacterial species, assimilation and utilization of nitrogen are challenges. Nitrogen metabolisms in most bacteria are regulated at transcription levels by a global transcriptional regulator that shows a great difference in Gram-negative and Gram-positive bacteria [1–3]. GlnR in the MerR family is one such important transcriptional regulator that regulates the global nitrogen metabolism in several low-GC Gram-positive bacteria, including *Bacillus subtilis* [4], *Lactococcus lactis* [5], and *Streptococcus pneumonia* [6].

The regulatory function of GlnR is well characterized in *B. subtilis*, where GlnR regulates the global nitrogen metabolism according to nitrogen availability together with TnrA [7]. GlnR and TnrA identify and bind to a common consensus DNA sequence (TGTNAN7TNACA) (GlnR/TnrA-sites), and regulate the nitrogen metabolism in *B. subtilis* by activating or regressing the relevant genes’ transcription through binding to these sites in their promoters [8]. GlnR represses gene transcription under excess nitrogen conditions, while TnrA regulates that when nitrogen is limited [9, 10]. The *glnR* gene (encoding GlnR) and *glnA* gene (encoding glutamine synthase, GS) constitute the operon *glnRA* [10]. In *B. subtilis*, GlnR negatively regulates the transcriptions of a series of genes/operons, including the *glnRA* operon (negative auto-regulation), *tnrA* [11], and the urease gene cluster (*ureABC*) [12] under excess nitrogen conditions. Meanwhile, TnrA regulates more genes/operons than GlnR under limited nitrogen conditions [13]. For example, TnrA activates *amtB-glnK* (encoding ammonium transporter AmtB and regulatory protein GlnK) [14], *nasA* and *nasBC/DEF* (encoding proteins related to nitrite reduction) [15], *pucR* (encoding purine catabolism regulator) [16] and its own gene *tnrA* [4], while it represses the *glnRA* operon [9], *alsT* (encoding an H^+^/Na^+^ amino acid symporter) [13] and *gltAB* (encoding glutamate synthase) [17, 18]. Under excess nitrogen conditions, the increasing glutamine content in the cells inhibits the activity of GS by turning it into feedback-inhibited GS (FBI-GS) [19]. The FBI-GS can act as a chaperone to stabilize GlnR-DNA complexes [7], but interact with TnrA physically to inhibit its DNA-binding activity [20].

*Paenibacillus* is a genus of facultative anaerobic and endospore-forming bacteria, which is Gram-positive, and shares a lot in common with the genus *Bacillus* [21]. *P. polymyxa* is an important species in the genus *Paenibacillus*, which has many plant-growth-promoting strains that could be used as bacterial fertilizer in agriculture. Our previous studies have demonstrated that *Paenibacillus polymyxa* WLY78 possesses several plant-growth-promoting traits [22] and a *nif* operon [23, 24]. Unlike *B. subtilis*, *P. polymyxa* WLY78 lacks *tnrA* and possesses only a *glnR* gene in its genome. Our recent study has revealed that GlnR positively and negatively regulates the *nif* genes’ expression by binding to the two GlnR-binding sites in the single *nif* promoter region according to nitrogen availability in this bacterium [25]. However, further identification of the genes directly regulated by GlnR is still required to demonstrate its global regulatory role in this bacterium.

Here, the potential GlnR DNA-binding sites were predicted at the genome scale, and verified by chromatin immunoprecipitation-quantitative PCR (ChIP-qPCR) and electrophoretic mobility shift assays (EMSA) in vivo and in vitro, respectively. Furthermore, genome-wide transcription analyses of wild-type (WT) and Δ*glnR* mutant grown anaerobically under nitrogen-limited and nitrogen-excess conditions were performed to investigate the differentially expressed genes. This study reveals that GlnR directly controls the expression of 17 genes/operons (a *nif* operon (*nifBHDKENXhesAnifV*), *glnRA*, *amtBglnK*, *glnA1*, *glnK1*, *glnQHMP*, *nasA*, *nasD1*, *nasD2EF*, *gcvH*, *ansZ*, *pucR*, *oppABC*, *appABCDF*, *dppABC*, *ldh3* and *maeA1*). These genes/operons fulfill three criteria: (1) the presence of a potential GlnR-binding site; (2) promoter region bound by GlnR in vivo; (3) GlnR-dependent expression regulation. Also, GlnR indirectly regulates the transcription of genes/operons involved in the Fe-S cluster synthesis, ferric transport and molybdenum transport that are specifically required for nitrogen fixation and the genes/operons involved in nitrogen and carbon metabolisms. This study demonstrates that GlnR has a multiple regulatory role in *P. polymyxa* WLY78.

## Results

### Prediction of GlnR target genes in the genome of *P. polymyxa* WLY78

For the prediction of GlnR-binding site, the promoters of each gene in the *P. polymyxa* WLY78 genome (bioproject accession number: PRJNA171204), which was defined as the regions from − 350 to + 50 bp relative to the translational start codon, were extracted (Additional file 1). These promoter sequences were scanned with the known GlnR-binding sequence in *Bacillus* (5′-TGTNA-N7-TNACA-3′) [8] using the MEME-MAST tool (version 4.11, http://meme-suite.org) [26] with default options.

A total of 488 putative GlnR-binding sites with *p* value ≤10^− 4^ were identified (Additional file 2). Twenty-four genes/operons with combined *p* value ≤0.05 and did not co-translate after other genes were identified as putative GlnR targets (Table 1). These genes/operons include a *nif* operon (*nifBHDKENXhesAnifV*) that is involved in nitrogen fixation, 15 genes/operons that are involved in nitrogen metabolism, 6 genes that are involved in carbon metabolism, and 2 other genes. Among the 24 putative target genes/operons, 14 of them (e.g., *glnRA, nasD2EF*) have one GlnR-binding site, and 10 (e.g., *nif* operon, *glnA1, amtBglnK, glnQHMP, nasD1,* and *ldh3*) have two GlnR-binding sites in their promoter regions. The genes *nasA* and *nasD1* share the same GlnR-binding sites as they are next to each other with a 353-bp spacing and have opposite transcription directions. The data suggest that GlnR may regulate these genes’ expression by binding to the GlnR-binding sites.

**Table 1:** The predicted GlnR-binding site(s) in the promoter regions of putative GlnR target genes in *P. polymyxa* WLY78

Gene name	Function	Co-trans gene	Putative binding site	***p*** value
*nifB*	nitrogenase cofactor biosynthesis protein	*nifHDKENXhesAnifV*	ACGATATATTACTTGACGT	1.60E-05
			ATGTAAGGGAATATAACGT	2.30E-05
*glnR*	MerR family transcriptional regulator	*glnA*	TTGTGAGTTTTTCTAACAT	1.50E-07
*glnA1*	glutamine synthetase		GCGTAAGCATATCTAACAT	2.80E-06
			TTGAGATTTATAATGACGA	5.10E-05
*amtB*	ammonium transporter	*glnK2*	TAGTTAGTAAATATGACAT	2.90E-05
			ATGTAAAGAAACTTAACAT	2.30E-07
*glnK1*	signal transduction histidine kinase		CTGTGAATTTAGCTCACAT	1.20E-05
*glnQ*	glutamine transport ATP-binding protein	*glnHMP*	ATGTGAAATTTAATAACAT	2.30E-06
			ACGTGAAGTTTTATGACAT	4.20E-07
*nasA*	nitrate transporter		ATGTCAACATTCAATACATG	5.90E-05
			GTGTTATGTATATTGACAT	7.10E-06
*nasD1*	nitrite reductase		ATGTCAATATACATAACAC	7.10E-06
			ATGTATTGAATGTTGACAT	5.90E-05
*nasD2*	nitrite reductase	*nasEF*	GCGTAAGGAATGTTGACAT	1.60E-06
*metN*	methionine ABC transporter ATP-binding protein	*metI*	TCGACATATTTCATACCAT	1.50E-05
*ansZ*	L-asparaginase		AAGTTATATTTTCTAACAT	1.10E-05
			TTGTCAGTTAATCTATCAT	4.10E-06
*gcvH*	glycine cleavage system protein H		ATGTCAATAAAAATGTCAT	1.10E-05
			TTGTTATAAATTATTATAT	8.70E-05
*pucR*	purine catabolism regulatory protein		ATGTCATGTAAAATGACAT	3.90E-07
*dppA*	peptide ABC transporter substrate-binding protein	*dppBC*	TTGTGAGTATATGTCACAC	2.00E-05
*oppA*	oligopeptide ABC transporter substrate-binding protein	*oppBC*	ATGTGATATAACATGACAT	1.90E-07
*appA*	oligopeptide ABC transporter substrate-binding protein	*appBCDF*	ACGTGATATTATTTTACAC	9.60E-06
*ldh3*	lactate dehydrogenase		ATGGCACTATTCCTTCCAT	8.70E-05
			ATGTTATGTTATGTAACGT	4.60E-07
*maeA1*	NAD-dependent malic enzyme		ATGTCATCTTTCGTTATGA	9.60E-06
*dxs*	1-deoxy-D-xylulose-5-phosphate synthase		TTGTTAACTTTACTCACAA	9.30E-07
*bglA1*	6-phospho-beta-glucosidase	*bglP*	TCGTTACGAATACTTACAG	8.30E-06
*bglA2*	6-phospho-beta-glucosidase		TTAAAATAAAACTTGACAT	1.30E-05
*gpsA*	glycerol-3-phosphate dehydrogenase		TTGTAATGACTCGTTACGTA	1.50E-05
*yvqE*	two-component sensor protein	yvqC	ACGTCATAAATTCTGACAT	3.50E-07
			ATGTTATAAAAAGTACCAA	4.00E-05
*sigF*	RNA polymerase sigma 70		CCGTCAGGTGTCGTAACGA	1.70E-05

### Identification of GlnR-regulated genes by ChIP-qPCR and EMSA

**Fig. 1:**
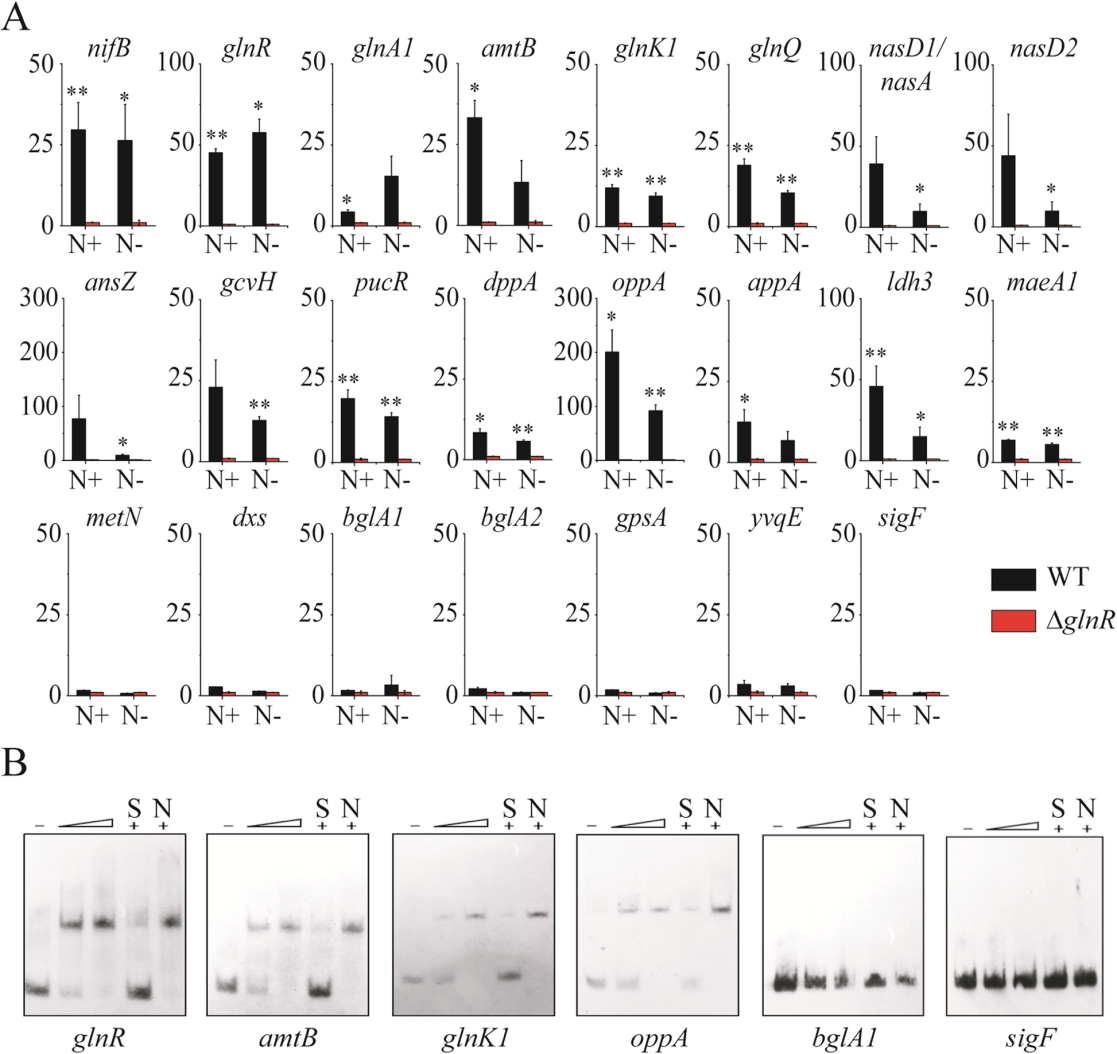
Validation of putative genes predicted to be directly regulated by GlnR. **A** The relative binding levels of GlnR on the upstream regions of putative genes determined by ChIP-qPCR. The binding levels in Δ*glnR* were arbitrarily set to 1.0. N+ and N- indicate nitrogen excess and limited condition, respectively. Error bars indicate SD from three independent experiments. **p < 0.01; * < 0.05. **B** EMSA of upstream regions of selected genes. Each lane contained 0.3 nM labeled probe. Lanes 2 to 5 contained 0.5, 1, 1, and 1 μM His_6_-GlnR respectively. Lanes -: EMSAs without His_6_-GlnR. A 200-fold excess of unlabelled specific probe (lanes S) or nonspecific competitor DNA (Probe 1) (lanes N) was used in competition assays. The original blots are shown in Fig. S1

As shown in Fig. 1A, ChIP-qPCR was used to investigate the accuracy of the predicted GlnR-binding sites in the 24 putative genes/operons described above. ChIP-qPCR revealed that GlnR significantly (*p* < 0.05) bound to the promoter regions of the *nif* operon, *glnRA*, *glnK1*, *glnQHMP*, *pucR*, *dppABC*, *oppABC*, *ldh3*, and *maeA1* under both conditions, the promoters of *glnA1*, *amtBglnK*, and *appABCDF* under the nitrogen-excess condition, and the promoters of *nasA*, *nasD1*, *nasD2EF*, *ansZ*, and *gcvH* under the nitrogen-limited condition. However, although putative binding sites were found in the promoter regions of *metN, dxs, bglA1, bglA2, gpsA, yvqEC* and *sigF*, their binding with GlnR was not detected. Among the 17 genes/operons that showed significant affinity with GlnR in their promoter regions, 15 belong to nitrogen fixation (the *nif* operon) and nitrogen metabolism (*glnRA*, *glnA1*, *amtBglnK*, *glnK1*, *glnQHMP*, *nasA*, *nasD1*, *nasD2EF*, *ansZ*, *gcvH*, *pucR*, *dppABC*, *oppABC*, *appABCDF*), and *2* belong to carbon metabolism (*ldh3* and *maeA1*).

The results of ChIP-qPCR were further examined using EMSA, where 6 promoters were selected to investigate their interaction with the purified His-tagged GlnR (Fig. 1B). It showed that GlnR bound to the promoter regions of *glnRA*, *amtBglnK*, *glnK1*, and *oppA*, but it did not bind to that of *bglA1* and *sigF*. These results were consistent with and confirmed the findings in ChIP-qPCR.

### Transcriptome analyses of wild-type and Δ*glnR* mutant strains

**Fig. 2:**
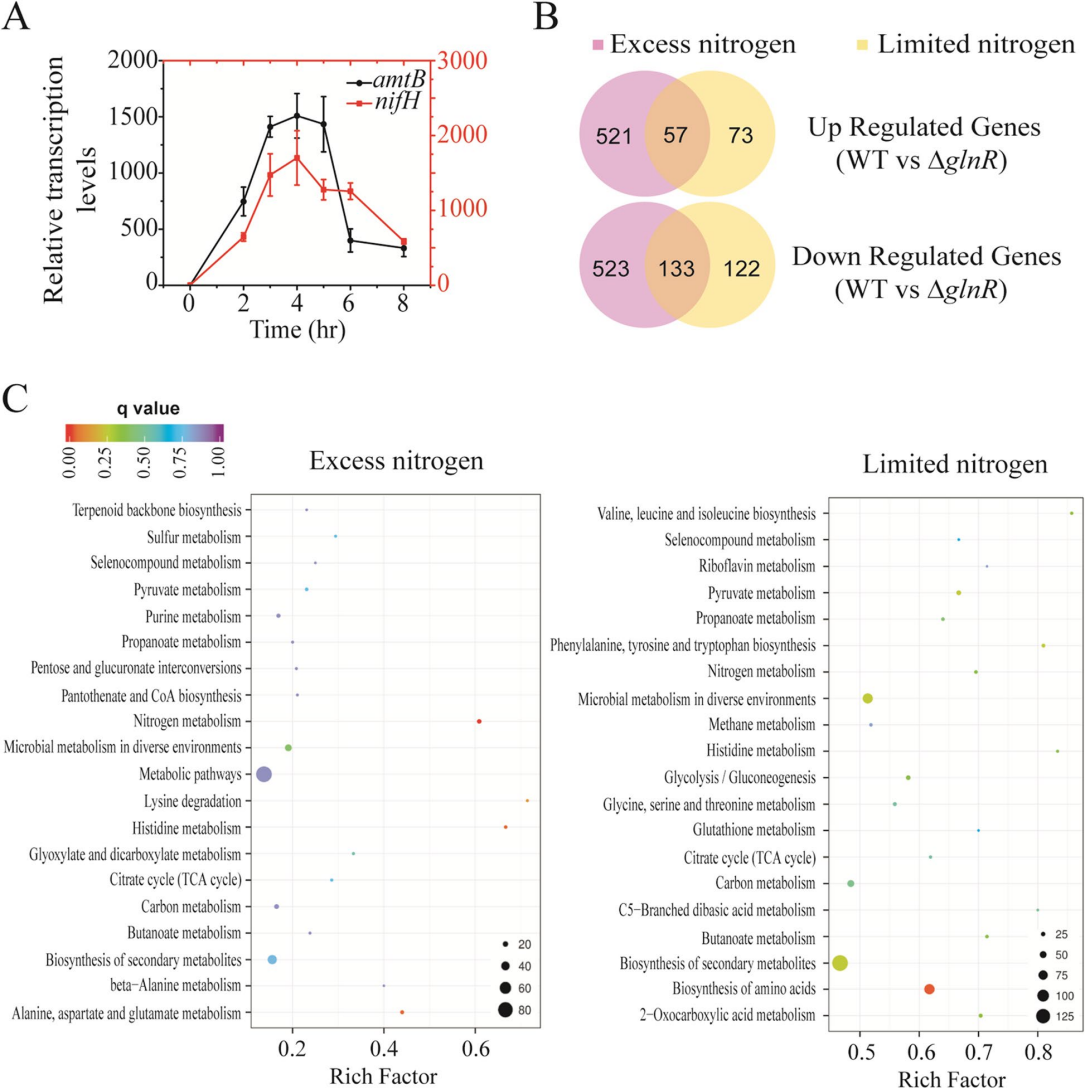
Transcriptome analyses of WT and Δ*glnR* grown under nitrogen-excess and -limited conditions. **A** Relative transcription levels of *amtB* and *nifH* in *P. polymyxa* WLY78 during anaerobic nitrogen-limited growth. Error bars indicate SD from three independent experiments. **B** Venn diagrams representing the differentially expressed genes under nitrogen-excess and nitrogen-limited conditions. **C** Statistics of KEGG pathway enrichment. The most enriched 20 pathways are shown

In order to elucidate the effects of GlnR protein on gene transcription in *P. polymyxa* WLY78, transcriptome analysis of WT and Δ*glnR* mutant strains grown under nitrogen excess and limited conditions were carried out respectively. The optimal cultivation period for global transcriptional analysis was 4 hours after the strains were transferred into the nitrogen excess or limited mediums as determined by the transcription levels of *amtB* and *nifH* in the wild-type (WT) strain cultured anaerobically under nitrogen-limited condition (Fig. 2A). Genes showing significant differences (adjusted *p* ≤ 0.05) with a |log2 fold change (FC)| ≥ 1 were identified as differential expression genes. A total of 190 genes were differentially expressed in WT under both conditions compared to the Δ*glnR* mutant. Thereinto, 57 genes were upregulated and 133 genes were downregulated in WT compared to Δ*glnR* mutant under both conditions (Fig. 2B). Compared to Δ*glnR,* the transcription of 578 genes were upregulated, and 656 genes were downregulated under excess nitrogen condition, while 130 were upregulated and 255 were down-regulated under nitrogen-limitation condition in WT. These results demonstrated that GlnR played a global regulatory role under not only nitrogen-excess, but also nitrogen-limited conditions. Moreover, GlnR could serve as both a repressor and an activator.

The pathways that the differentially expressed genes belong to were determined using KEGG enrichment analysis. As shown in Fig. 2C, the genes under the control of GlnR are mainly involved in nitrogen fixation, nitrogen metabolism, biosynthesis of amino acids, amino acids metabolism, citrate cycle (TCA cycle), carbon metabolism, sulfur metabolism and biosynthesis of secondary metabolites. These results imply that GlnR plays an important role in regulating not only nitrogen assimilation, but also other important pathways, especially carbon metabolism. Thus, the transcription level of these differentially expressed genes involved in nitrogen fixation, nitrogen metabolism and carbon metabolism are individually analyzed as follows.

### GlnR regulates the transcription of *nif* and the non-*nif* genes required in nitrogen fixation

**Fig. 3:**
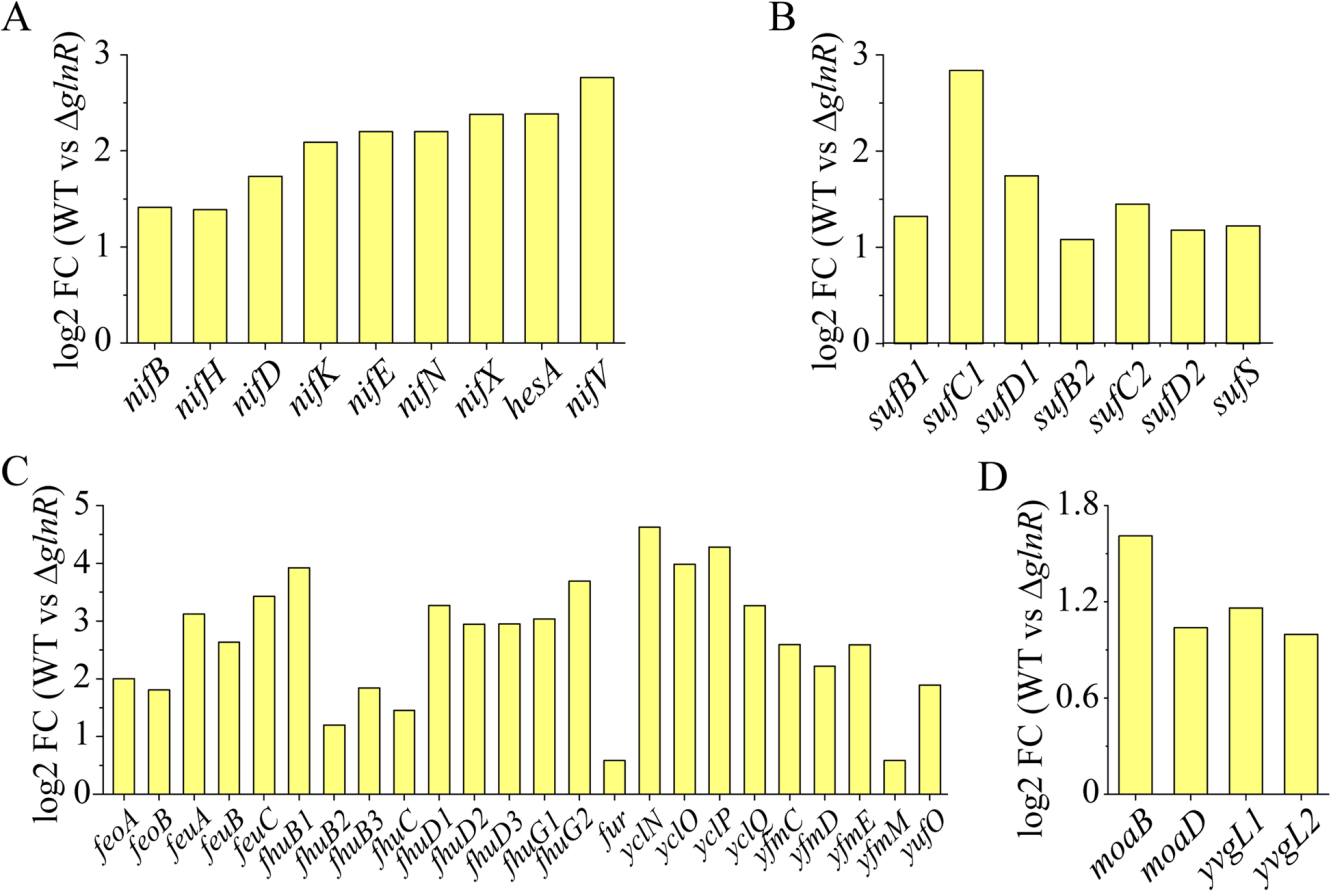
GlnR affects the transcription of *nif* genes and the non-*nif* genes required for nitrogen fixation under nitrogen-limited condition. **A** Differential expression of *nif* genes under nitrogen-limited condition. **B** Differential expression of genes involved in metabolism of sulfate under nitrogen-limited condition. **C** Differential expression of genes involved in transporting of iron under nitrogen-limited condition. **D** Differential expression of genes involved in the metabolism of molybdenum under nitrogen-limited condition

KEGG enrichment analysis revealed that not only the *nif* operon, but also the genes required for nitrogen fixation were differentially expressed under nitrogen-limited conditions. The 9 genes (*nifB, nifH, nifD, nifK, nifE, nifN, nifX, hesA, nifV*) within the *nif* operon showed increases ranging from 1.4- to 2.8- log2(FC) in WT compared with Δ*glnR* mutant under nitrogen-limited (nitrogen-fixing) condition (Fig. 3A; Table S2), supporting that GlnR directly and positively regulates *nif* gene transcription [25]. Moreover, the non-*nif* genes required in nitrogen fixation were coordinately regulated by GlnR. For example, the genes (e.g., *sufBCD*, *sufS*) involved in the synthesis of Fe-S cluster were up-regulated (Fig. 3B). The genes (e.g., *feoAB*, *feuABC*, *fhuB*, *fhuC*, *fhuD*, *fhuG*, *yclNOPQ* and *yfmCDE*) involved in ferric transport were up-regulated (Fig. 3C). Four genes (*yvgL1, yvgL2, moaB* and *moaD*) involved in molybdenum transport and metabolism were up-regulated in WT compared with Δ*glnR* under nitrogen-limited condition (Fig. 3D). Since no GlnR-binding site was found in the promoters of these genes, we deduce that GlnR indirectly regulates the expression of these non-*nif* genes that required for nitrogen fixation.

### GlnR regulates the transcription of the genes involved in nitrogen assimilation

**Fig. 4:**
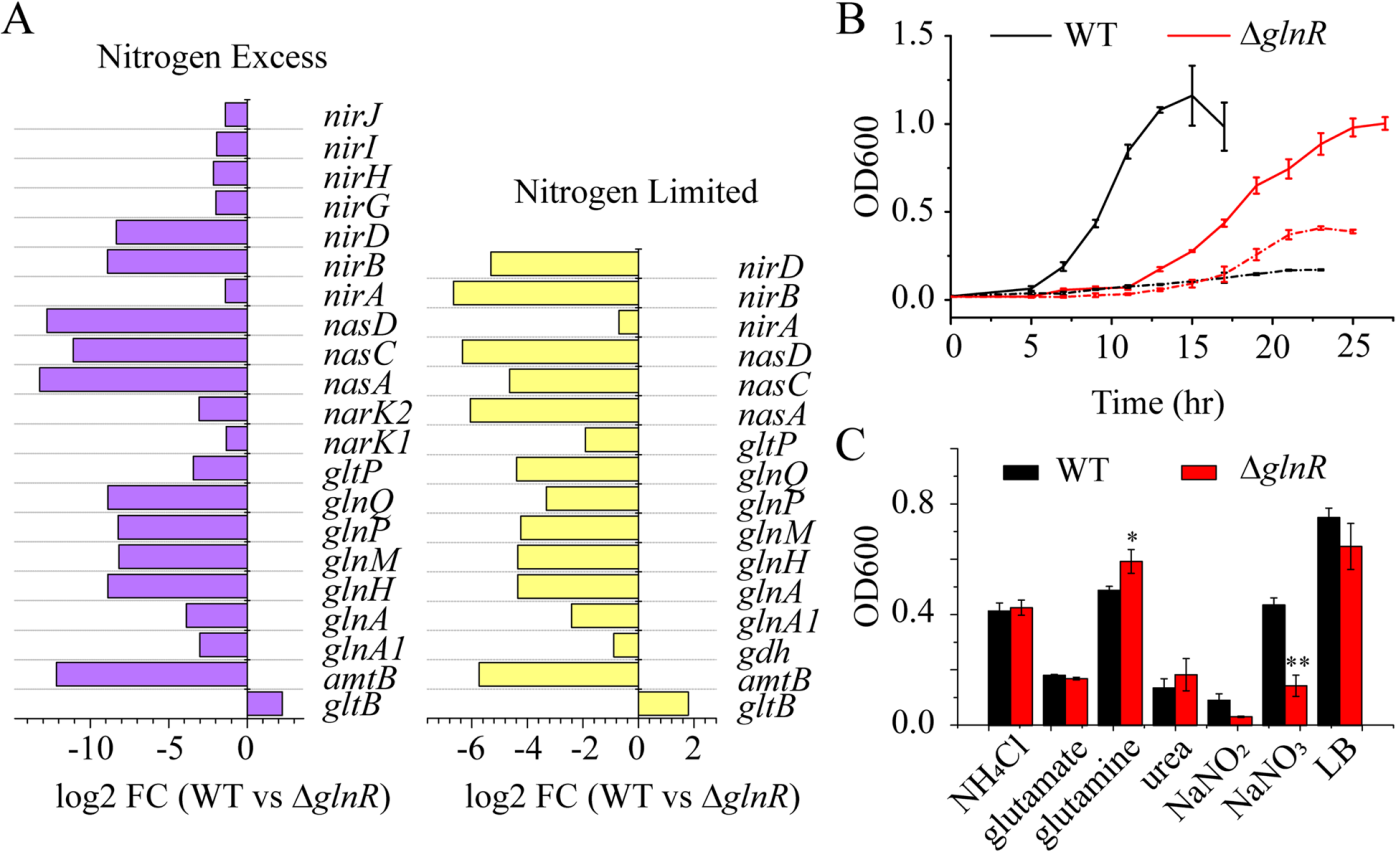
Different nitrogen-metabolism-involved gene expression and nitrogen source utilization of WT and Δ*glnR*. **A** Differential expression of genes involved in nitrogen metabolism determined by KEGG enrichment. **B** Growth curve of WT and Δ*glnR*. Solid line represents the growth under nitrogen-excess condition; dash line represents the growth under nitrogen-limited condition. Error bars indicate SD from three independent experiments. **C** Growth status of WT and Δ*glnR* in minimal medium with 30 mM NH_4_Cl, glutamate, glutamine, urea, NaNO_2_, or NaNO_3_ as sole nitrogen source. Error bars indicate SD from three independent experiments. **p < 0.01; *p < 0.05

KEGG enrichment analysis revealed that the genes involved in nitrogen metabolism were differentially expressed (Fig. 2C, Fig. 4A). When compared with Δ*glnR* mutant, both *glnA* within *glnRA* operon and *glnA1* encoding glutamine synthase (GS), *amtB*, *nasA*, *nasDC*, *narGHJI*, *narK1K2*, *nirABD*, *glnQHMP*, and *gltP* were down-regulated in WT under both nitrogen-limited and -excess conditions. The *gltB* encoding glutamate synthase (GOGAT) was up-regulated in WT compared with Δ*glnR* mutant under both conditions. Also, *gdh* encoding glutamate dehydrogenase was down-regulated in WT compared with Δ*glnR* mutant under nitrogen-limited condition. The *nirGHIJ* were down-regulated in WT compared with Δ*glnR* mutant in nitrogen-excess condition. As described above, there are GlnR-binding site(s) in the promoter regions of *glnRA*, *glnA1, amtBglnK*, *nasA, nasDC, glnQHMP,* indicating that GlnR directly and negatively controls the expression of these genes involved in nitrogen metabolism. However, no GlnR-binding site was found in the promoter regions of *gdh, gltP, narK1K2*, *nirABD*, and *gltB*, suggesting that GlnR indirectly controls the expression of these genes involved in nitrogen metabolism.

To test the role of GlnR in nitrogen utilization in *P. polymyxa* WLY78, growth tests with different nitrogen sources were carried out. When grown under nitrogen-excess condition, WT got into the logarithmic phase earlier than Δ*glnR* strain, but both of their growth came to a stop at a similar OD_600_ value. Both strains grew poorly under nitrogen-limited condition, but Δ*glnR* mutant was less affected (Fig. 4B). When grown 2 days in a minimal medium with 30 mM of different unique nitrogen sources, WT and Δ*glnR* strains showed similar growth levels on NH_4_Cl and glutamate, growth levels of WT were higher than those of Δ*glnR* mutant on NaNO_2_ and NaNO_3_, but lower on glutamine and urea (Fig. 4C), consistent with the reports that GlnR represses expression of *glnA* and *ureABC* in *B. subtilis* [11, 12].

### GlnR regulates the transcription of genes involved in carbon metabolism directly and indirectly

**Fig. 5:**
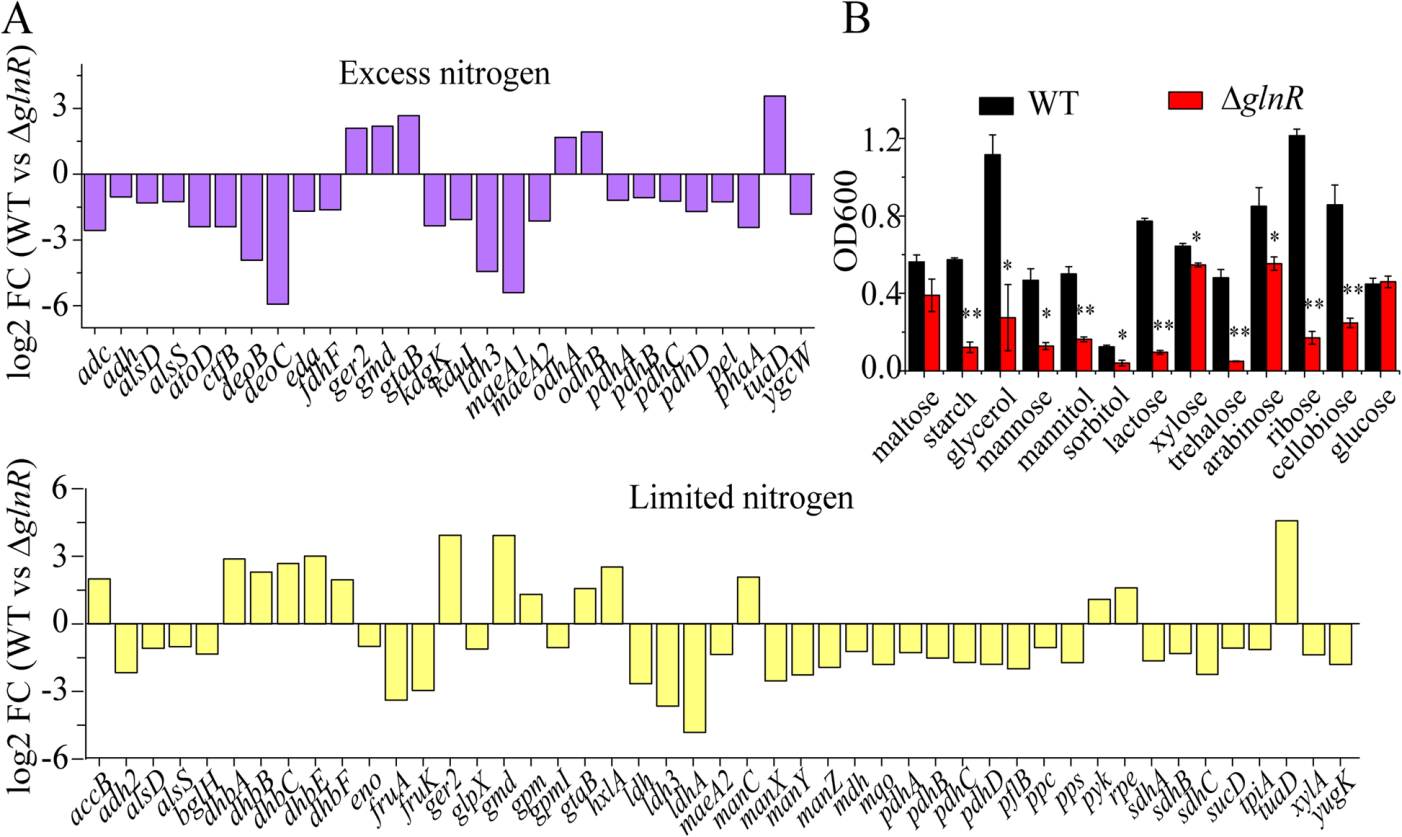
Different carbon-metabolism-involved gene expression and carbon source utilization of WT and Δ*glnR*. **A** Differential expression of genes involved in carbon metabolism. **B** Growth status of WT and Δ*glnR* in minimal medium with 0.4% maltose, starch, glycerol, mannose, mannitol, sorbitol, lactose, xylose, trehalose, arabinose, ribose, cellobiose, and glucose as sole carbon source. Error bars indicate SD from three independent experiments. **p < 0.01; *p < 0.05

KEGG enrichment analysis revealed that there were 16 and 47 genes involved in carbon metabolism were affected by GlnR under nitrogen-excess and -limited conditions, respectively (Fig. 2C, Table S3). Among these genes, *ldh3* (lactate dehydrogenase) and *maeA1* (malic enzyme) have GlnR-binding site(s) in their promoter regions. But there is no GlnR-binding site in the promoter regions of other genes, including the genes involved in TCA cycle (*odhAB*, *pdhABCD*), pentose phosphate pathway (*kdgK*, *deoBC*), pyruvate metabolism (*pyk*), xylose isomerase (*xylA*), mannose metabolism (*manC*, *manXYZ*) and succinate dehydrogenase (*sdhABC)* (Fig. 5A). Growth tests showed that Δ*glnR* mutant was deficient in utilizing starch, glycerol, mannose, mannitol, sorbitol, lactose, xylose, trehalose, arabinose, ribose, and cellobiose (Fig. 5B). These results indicated that GlnR controls carbon metabolism directly and indirectly.

## Discussion

GlnR is characterized as an important regulator in nitrogen metabolism, which regulates genes through binding to the consensus GlnR-binding sites in promoters [7, 8]. In the facultative anaerobic and nitrogen-fixing strain *P. popymyxa* WLY78, GlnR has been found to activate the transcription of *nif *genes under nitrogen-limited condition, while repress that under nitrogen-excess condition in our previous work [25]. In this study, a global identification of the genes directly and indirectly regulated by GlnR is determined by using ChIP-qPCR, EMSA and genome-wide transcriptional analysis in *P. polymyxa* WLY78. Our results demonstrate that GlnR is not only involved in the regulation of nitrogen fixation and nitrogen metabolism, but also in carbon assimilation. Seventeen genes/operons are identified as directly regulated by GlnR, fifteen of which are the newly identified targets of GlnR.

The 17 genes/operons that are directly regulated by GlnR include a *nif* operon (*nifBHDKENXhesAnifV*) for nitrogen fixation, 14 nitrogen metabolism genes (*glnRA*, *glnA1*, *amtBglnK*, *glnK1*, *glnQHMP*, *nasA*, *nasD1*, *nasD2EF*, *ansZ*, *gcvH*, *pucR, oppABC*, *appABCDF*, *dppABC*) and *2* carbon metabolism genes (*ldh3* and *maeA1*). They fulfill three criteria: (1) the presence of a GlnR-binding site; (2) GlnR binding in ChIP-qPCR experiments; (3) GlnR-dependent expression regulation, indicating that these genes/operons are directly regulated by GlnR. Our results that GlnR binds to the promoter regions of *nif* operon and *glnRA* are consistent with the reports that GlnR binds to the promoter regions of *nifB* and *glnRA* in *Paenibacillus riograndensis* SBR5T [27]. It is known that GlnR represses the transcription of the *glnRA*, *ureABC, tnrA,* and *alsT* in *B. subtilis* depending on the nutritional conditions [13, 28]. However, *alsT*, *tnrA* and *ureABC* were not found in the genome of *P. polymyxa* WLY78.

Our results reveal that GlnR regulates the expressions of the 8 genes/operons (*amtBglnK, glnQHMP, pucR, nasA, nasD2EF, oppABC, appABCDF* and *ansZ*) in *P. polymyxa* WLY78, which are controlled by TnrA in *B. subtilis* [13, 29]. The data suggest that GlnR has undertaken the roles of both GlnR and TnrA [28, 29]. GlnR with functions of GlnR and TnrA in *P. polymyxa* WLY78 agrees with the facts that GlnR and TnrA in *B. subtilis* are similar in the protein sequences in their N terminal domains, and bind to a common consensus sequence (5′-TGTNAN7TNACA-3′) [8, 11, 30]. In addition, this study has revealed that *glnK1*, *glnA1*, *gcvH* and *dppABC* involved in nitrogen metabolism and *ldh3* and *maeA1* involved in carbon metabolism are new targets regulated by GlnR in *P. polymyxa* WLY78. Especially, we have identified *dppABC*, *gcvH*, *maeA1 and ldh3* as the new members of the GlnR regulon in *P. polymyxa* WLY78, since expressions of these genes/operons under control of GlnR or TnrA in *B. subtilis* have never been reported.

The transcriptome profile has shown that GlnR not only directly regulates the transcription of the *nif* genes, but also indirectly regulates the expression of genes (encoding transporters of Fe, S and Mo atoms) specifically required in nitrogen fixation. The results indicate that GlnR controls the process of nitrogen fixation not only by regulating *nif* operon at the transcription level, but also by affecting the transportation of the essential genes for nitrogenase synthesis.

GlnR is the global nitrogen regulator in the class *Bacilli*, which plays an important role in regulating nitrogen utilization and assimilation. In this study, transcriptional analyses reveal that *P. polymyxa* GlnR negatively regulates the expression of *glnA* within *glnRA* operon, *glnA1*, *amtB*, *nasA*, *nasD*, and *glnQHMP*, whose promoter regions have GlnR-binding site(s). Also, GlnR negatively regulates the expression of *narGHJI*, *narK1K2*, *nirABD, nirGHIJ*, *gltP* and *gdh*, whose promoter regions have no GlnR-binding site(s). GlnR activates the expression of *gltB* which does not have a GlnR-binding site in its promoter region. However, in *B. subtilis* TnrA negatively controls the expression of *gltAB* that has a TnrA-binding site in its promoter [18]. Although *ureABC* operon is not identified in *P. polymyxa* WLY78, the growth level of Δ*glnR* mutant was higher than that of WT on urea, suggesting that GlnR is involved in urea utilization. These results indicate that GlnR regulates the expression of genes involved in nitrogen metabolism directly and indirectly.

Here, we have shown that GlnR directly regulates the expression of *ldh3* (lactate dehydrogenase) and *maeA1* (malic enzyme) that have GlnR-binding site(s) in their promoter regions. Both *ldh3* and *maeA1* involved in carbon metabolism are newly identified targets of GlnR. Moreover, it is found that GlnR indirectly regulates the expression of the genes involved in carbon metabolism, since these genes (e.g., *xylA, odhAB*, *pdhABCD, kdgK*, *deoBC*, *sdhABC*) involved in carbon metabolism have no GlnR-binding site in their promoter regions. Also, Δ*glnR* strain showed growth defects when starch, glycerol, mannose, lactose, xylose, trehalose, arabinose, ribose, and cellobiose were used as sole carbon sources, supporting that GlnR is involved in carbon metabolism. It is reported that GlnR is involved in carbon utilization in actinomycetes [31].

**Fig. 6:**
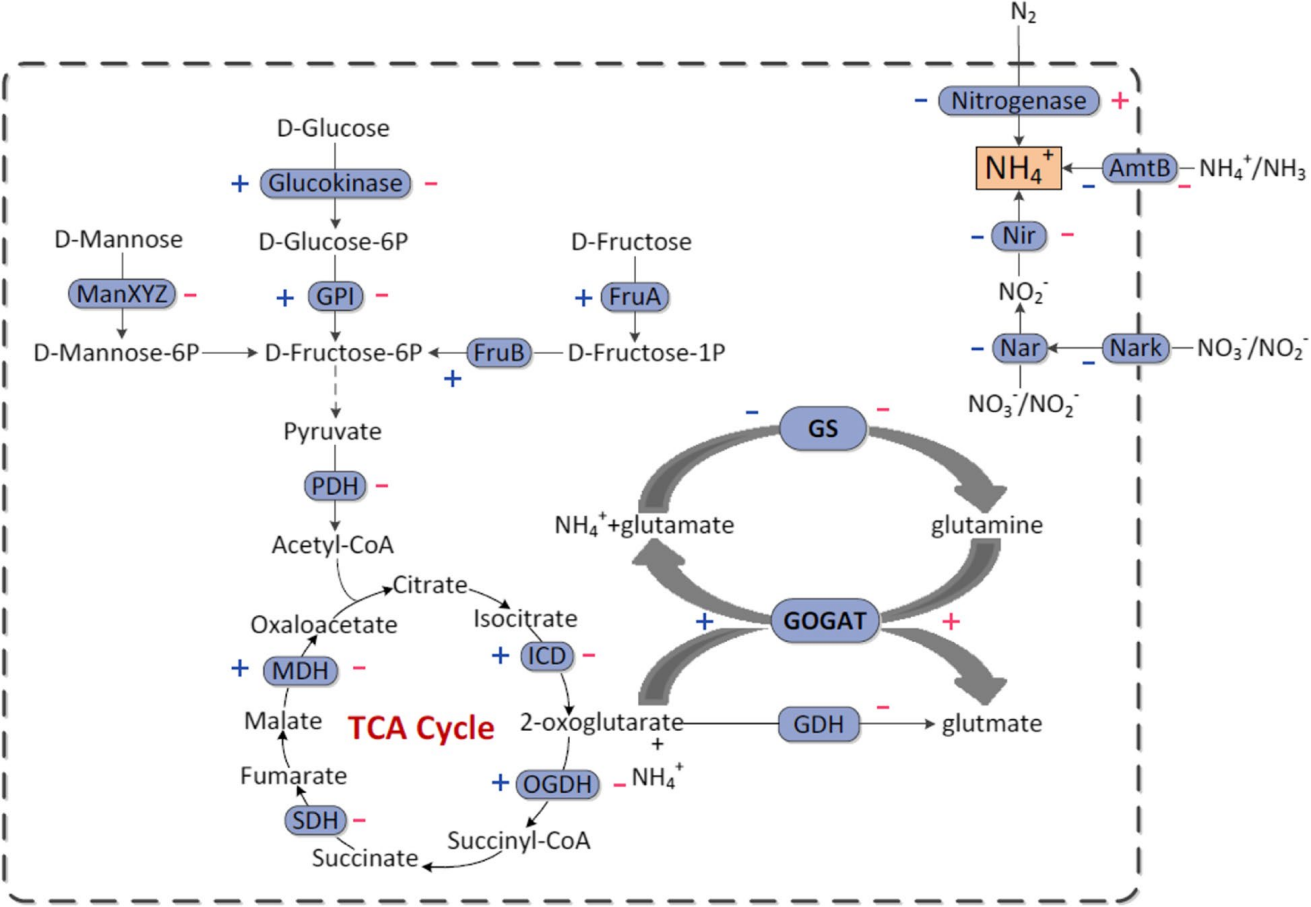
GlnR mediates a regulatory network of nitrogen and carbon metabolism in *P. polymyxa* WLY78. + and – in blue and red indicate the genes encoding the enzymes are upregulated and downregulated under nitrogen excess and limited condition, respectively. GPI (glucose phosphate isomerase) and PDH (pyruvate dehydrogenase), ICD (isocitrate dehydrogenase), OGDH (ketoglutarate dehydrogenase), SDH (succinate dehydrogenase), MDH (malate dehydrogenase), GS (glutamine synthetase), GOGAT (glutamate synthetase), GDH (glutamate dehydrogenase), NIR (nitrite reductase) and NAR (nitrate reductase). Metabolism pathways presented are modified from KEGG pathway database [32, 33]

Taken together, this study has revealed that GlnR of *P. polymyxa* WLY78 is a global transcriptional regulator which controls the expression of genes involved in nitrogen fixation, nitrogen metabolism and carbon metabolism (Fig. 6). Among these genes, some are directly regulated by GlnR and some are indirectly regulated by GlnR.

## Conclusions

This study reveals that GlnR directly regulates the expression of 17 genes/operons whose promoter regions have GlnR-binding sites. These genes/operons include a *nif* operon (*nifBHDKENXhesAnifV*) for nitrogen fixation, 14 nitrogen metabolism genes/operons (*glnRA*, *amtBglnK*, *glnA1*, *glnK1*, *glnQHMP*, *nasA*, *nasD1*, *nasD2EF*, *gcvH*, *ansZ*, *pucR*, *oppABC*, *appABCDF* and *dppABC)* and 2 carbon metabolism genes (*ldh3* and *maeA1*)*.* Except for *glnRA* and *nif operon*, the other 15 genes/operons are newly identified targets regulated by GlnR. This is the first time to find that GlnR directly regulates the expression of *maeA1* and *ldh3* involved in carbon catabolism. Furthermore, our study has shown that GlnR indirectly controls transcription of the non-*nif* genes specifically required for nitrogen fixation and the genes involved in nitrogen and carbon metabolism. This study demonstrates that GlnR is a global regulator controlling the expression of the genes involved in nitrogen fixation and nitrogen and carbon metabolisms in low-GC Gram-positive bacterium *P. polymyxa* WLY78.

## Methods

### Bacterial strains and growth conditions

*Paenibacillus polymyxa* WLY78 was the wild-type strain used throughout this study. Mutant Δ*glnR* was constructed by an in-frame deletion of *glnR* gene in *P. polymyxa* WLY78 [25]. *P. polymyxa* strains were cultured at 30 °C, in LD medium for routine culture, in nitrogen-deficient media for nitrogen-limited condition, and nitrogen-deficient media with 100 mM NH_4_Cl for nitrogen-excess condition. The LD and nitrogen-deficient media were prepared in the same way as our previous study [24]. *Escherichia coli* BL21 (DE3) was used to express His_6_-GlnR and grown in LB medium.

### Purification of recombinant GlnR

The His_6_-GlnR was overexpressed in *E. coli* BL21 (DE3) containing plasmid pETGlnR and purified as described by Wang, et al. [25]. The plasmid pETGlnR was constructed by cloning *glnR* gene into pET28b (Novagen). The recombinant *E. coli* strain was cultivated in LB broth at 37 °C till mid-log phase and then incubated with 0.5 mM IPTG at 20 °C to induce the expression of His_6_-GlnR. By sonication on ice, the collected recombinant *E. coli* strain cells were disrupted in a lysis buffer containing 10 mM Imidazole, 50 mM NaH_2_PO_4_, and 300 mM NaCl. After centrifuged, the supernatant was loaded into Ni^2+^-NTA resin (Qiagen, Germany), purified using lysis buffer containing 20 mM imidazole, and then the recombinant GlnR protein was eluted using lysis buffer containing 250 mM imidazole (Fig. S2). For the binding reactions in EMSA, the eluted His_6_-GlnR protein solution was dialyzed in a binding buffer, which contains 20 mM HEPES pH 7.6, 1 mM EDTA, 10 mM (NH_4_)_2_SO_4_, 1 mM DTT, 0.2% Tween 20, and 30 mM KCl. For the preparation of GlnR polyclonal rabbit antibody, the eluted His_6_-GlnR protein solution was dialyzed in a storage buffer, which contains 10 mM Tris-HCl pH 7.5, 1 mM EDTA, and 80 mM NaCl, 20%(v/v) glycerol. The titer of the antibody is 51,200 as identified by ELISA assay (Fig. S3).

### Chromatin immunoprecipitation-quantitative PCR (ChIP-qPCR)

The ChIP-qPCR was performed as described by Wang, et al. [25]. One hundred ml of culture were harvested after 4-hour growth under both nitrogen-limited and -excess conditions, respectively. The harvested cells were fixed with formaldehyde solution for 20 min at 28 °C, and the cross-linking was stopped by adding glycine to a final concentration of 125 mM. After washing with PBS buffer, 1 mg collected cells were sonicated on ice in 4 ml lysis buffer to break chromosomal DNA into 200–500 bp fragments. Precleared supernatant containing 2 mg protein in total was incubated with 5 μl GlnR polyclonal antibody overnight at 4 °C. After incubated with 50 μl Protein G Sepharose (GE, Sweden) for 4 hours at 4 °C, the immune complexes were purified and eluted from the beads. After reverse cross-linking and purification, immunoprecipitated DNA was used for qPCR template, and primers used for ChIP-qPCR are listed in Table S1. Relative binding levels of GlnR and the putative binding sites were determined by comparison with the control strain Δ*glnR*. The relative binding level was calculated as follows with the promoter region of 16S rRNA gene as control region:

$$\Delta \textrm{Ct}=\textrm{Ct}\ \left(\textrm{target}\ \textrm{region}\right)-\textrm{Ct}\ \left(\textrm{control}\ \textrm{region}\right)$$

$$\Delta \Delta \textrm{Ct}=\Delta \textrm{Ct}\ \left(\textrm{Wild}\ \textrm{type}\right)-\Delta\ \left(\Delta glnR\right)$$

### Electrophoretic mobility shift assays (EMSAs)

A DIG Gel Shift Kit (2nd Generation; Roche, USA) was used to perform EMSA following the manufacturer’s protocol. For promoters of *oppA*, *glnR*, *amtB*, and *glnK1*, the first strands of the fragments containing putative GlnR-binding sites and its complementary DNA strands were synthesized separately and annealed to form DNA probes (Table S1). For promoters of *glnQ* and *glnA1*, probes were amplified by PCR using certain primers listed in Table S1 and *P. polymyxa* WLY78 genome as template. The DNA fragments were labeled at the 3′ end with digoxigenin using terminal transferase, and then used as probes. The reaction systems were incubated at 25 °C for 30 min, analyzed by native 5% polyacrylamide gel in 0.5 × TBE buffer at 4 °C, and then transferred to a positively charged nylon membrane (GE Healthcare, UK). Labeled DNA on the membrane was detected by chemiluminescence, and recorded on X-ray film as described by Wang, et al. [24]. Each 20 μl reaction system contained 1 μg poly [d(A-T)], 0.3 nM labeled probe, and 0, 0.5, or 1 μM His6-GlnR. Reactions with no His_6_-GlnR were set as blanks. A 200-fold excess of unlabelled specific probe or nonspecific competitor DNA (Probe 1 in DIG Gel Shift Kit) was used in competition assays.

### RNA preparation and RNA-seq analysis

Total RNAs of the strains were extracted using RNAiso Plus (Takara, Japan) as described in the previous study [25]. The extracted RNA was used to synthesize cDNA after removing the residual genome DNA using PrimeScript™ RT reagent Kit with gDNA Eraser (Takara, Japan). qRT-PCR was conducted on Applied Biosystems 7500 Real-Time System (Life Technologies) to analyze the transcription levels of *nifH* and *amtB*, according to which the proper time for harvesting the *P. polymyxa* strains’ cells for transcriptome sequencing was determined. 16S rRNA gene was used as reference gene in qRT-PCR.

cDNA library was constructed and sequenced at Novogene Bioinformatics Institute (Beijing, China), and two biological replicates of each sample were carried out (available in the NCBI repository, bioproject accession number: PRJNA871995). The sequencing reads were mapped against the genome sequence of *P. polymyxa* WLY78 (PRJNA171204). Differential expression analysis was performed using the DESeq package (1.18.0) in R software. The *p*-values were adjusted using the Benjamini and Hochberg’s approach [34], and differentially expressed genes were assigned at adjusted *p*-value < 0.05 with |log 2(FC)| > 1. The entire expression data of all genes in each treatment is listed in Additional file 3. Kyoto Encyclopedia of Genes and Genomes (KEGG) pathway enrichment was analyzed by KOBAS (3.0) [35].

## Supplementary Information

**Additional file 1. **The promoter sequence set of *Paenibacillus polymyxa* WLY78.

**Additional file 2. **The GlnR putative binding sets in *Paenibacillus polymyxa* WLY78 identified by the MEME-MAST tool.

**Additional file 3. **The expression of genes in *Paenibacillus polymyxa* WLY78 in WT and delta *glnR* mutant under nitrogen excess and limited conditions. **Table S1.** Primers used in this study. **Table S2.** Transcription of genes involved in nitrogen fixation and metabolism. **Table S3.** Transcription of genes involved in carbon metabolism. **Fig. S1.** The original blots of EMSA results in Fig. 1B. Each lane contained 0.3 nM labeled probe. For the 5 lanes of the EMSA of each gene promoter, lanes 1 to 5 contained 0, 0.5, 1, 1, and 1 μM His_6_-GlnR respectively. A 200-fold excess of unlabelled specific probe (lanes 4) or nonspecific competitor DNA (Probe 1) (lanes 5) was used in competition assays. **Fig. S2.** The SDS-PAGE result of the purified His6-GlnR protein. The lanes from left to right are marker, solution eluted by lysis buffer containing 20 mM imidazole, solution eluted by 1 mL lysis buffer containing 250 mM imidazole from the first to the fourth time. **Fig. S3.** The titer of GlnR polyclonal antibody determined by ELISA.

## Data Availability

The dataset supporting the conclusions of this article is available in the NCBI repository, https://www.ncbi.nlm.nih.gov/bioproject/PRJNA871995.
