# Supplementary material for: Genome-wide mapping of GlnR-binding sites reveals the global regulatory role of GlnR in controlling the metabolism of nitrogen and carbon in Paenibacillus polymyxa WLY78
Source: BMC Genomics. 2023 Feb 23;24:85. doi: 10.1186/s12864-023-09147-1 (PMC9948412; doi:10.1186/s12864-023-09147-1)
Supplement: Supplementary file 3 — Additional file 3. The expression of genes in Paenibacillus polymyxa WLY78 in WT and delta glnR mutant under nitrogen excess and limited conditions. Table S1. Primers used in this study. Table S2. Transcription of genes involved in nitrogen fixation and metabolism. Table S3. Transcription of genes involved in carbon metabolism. Fig. S1. The original blots of EMSA results in Fig. 1B. Each lane contained 0.3 nM labeled probe. For the 5 lanes of the EMSA of each gene promoter, lanes 1 to 5 contained 0, 0.5, 1, 1, and 1 μM His6-GlnR respectively. A 200-fold excess of unlabelled specific probe (lanes 4) or nonspecific competitor DNA (Probe 1) (lanes 5) was used in competition assays. Fig. S2. The SDS-PAGE result of the purified His6-GlnR protein. The lanes from left to right are marker, solution eluted by lysis buffer containing 20 mM imidazole, solution eluted by 1 mL lysis buffer containing 250 mM imidazole from the first to the fourth time. Fig. S3. The titer of GlnR polyclonal antibody determined by ELISA. [file 12864_2023_9147_MOESM3_ESM.zip › Table S3.docx]

**Table S3. Transcription of genes involved in carbon metabolism**

| **Gene ID** | **Gene name** | **Gene product** | **log2FC**  **(WT vs Δ*glnR*)** | **log2FC**  **(WT vs Δ*glnR*)** |
| --- | --- | --- | --- | --- |
|  |  |  | **Nitrogen-excess** | **Nitrogen-limited** |
| **GM000072** | *adh* | ethanol-active dehydrogenase/acetaldehydc-active reductase | -1.0279 |  |
| **GM000198** | *eno* | enolase (2-phosphoglycerate dehydratase) | -1.0006 |  |
| **GM000199** | *gpmI* | phosphoglyceromutase |  | -1.0458 |
| **GM000476** | *mqo* | malate: quinone oxidoreductase | -1.0279 |  |
| **GM000554** | *ldhA* | lactate dehydrogenase |  | -4.8135 |
| **GM000674** | *hxlA* | 3-hexulose-6-phosphate synthase | 2.5237 |  |
| **GM000723** | *kdgK* | 2-dehydro-3-deoxygluconokinase | -2.3553 |  |
| **GM000725** | *kduI* | 5-keto-4-deoxyuronate isomerase | -2.0655 |  |
| **GM000726** | *eda* | ketohydroxyglutarate aldolase | -1.681 |  |
| **GM000727** | *ygcW* | 2-deoxy-D-gluconate 3-dehydrogenase | -1.8114 |  |
| **GM001138** | *ppc* | phosphoenolpyruvate carboxylase | -1.0494 |  |
| **GM001209** | *deoC* | deoxyribose-phosphate aldolase | -5.9257 |  |
| **GM001212** | *deoB* | phosphopentomutase | -3.9197 |  |
| **GM001285** | *xylA* | xylose isomerase |  | -1.3651 |
| **GM001401** | *bglH* | 6-phospho-beta-glucosidase |  | -1.3434 |
| **GM001489** | *sdhC* | succinate dehydrogenase |  | -2.2378 |
| **GM001490** | *sdhA* | succinate dehydrogenase |  | -1.6446 |
| **GM001491** | *sdhB* | succinate dehydrogenase |  | -1.3161 |
| **GM001635** | *maeA1* | malate dehydrogenase | -5.4017 | -2.5329 |
| **GM001655** | *yugK* | butanol dehydrogenase |  | -1.8025 |
| **GM002402** | *maeA* | malate dehydrogenase | -2.131 |  |
| **GM002477** | *rpe* | ribulose-phosphate 3-epimerase | 1.5957 |  |
| **GM002510** | *adh2* | acetaldehyde dehydrogenase |  | -2.1624 |
| **GM002511** | *pflB* | formate acetyltransferase |  | -1.994 |
| **GM002539** | *accB* | acetyl-CoA carboxylase |  | 1.9981 |
| **GM002717** | *fdhF* | formate dehydrogenase | -1.6222 |  |
| **GM003116** | *pdhA* | pyruvate dehydrogenase | -1.1864 | -1.28 |
| **GM003117** | *pdhB* | pyruvate dehydrogenase El component, beta subunit | -1.0609 | -1.5133 |
| **GM003118** | *pdhC* | dienelactone hydrolase | -1.234 | -1.7159 |
| **GM003119** | *pdhD* | dihydrolipoamide dehydrogenase | -1.6984 | -1.7874 |
| **GM003179** | *ldh* | lactate dehydrogenase |  | -2.654 |
| **GM003269** | *pps* | phosphoenolpyruvate synthase | -1.7245 |  |
| **GM003602** | *glpX* | fructose 1, 6-bisphosphatase | -1.1084 |  |
| **GM003731** | *alsS* | acetolactate synthase | -1.2474 | -1.0169 |
| **GM003732** | *alsD* | alpha-acetolactate decarboxylase | -1.3033 | -1.0853 |
| **GM003922** | *sucD* | succinyl-CoA synthetase subsunit alpha | -1.0733 |  |
| **GM004173** | *fruA* | PTS fructose transporter subunit IIC | -3.3888 |  |
| **GM004174** | *fruK* | phosphofructokinase |  | -2.9561 |
| **GM004203** | *mdh* | malate dehydrogenase |  | -1.2226 |
| **GM004215** | *pyk* | pyruvate kinase |  | 1.0846 |
| **GM004412** | *odhB* | dihydrolipoamide succinyltransferase | 1.9326 |  |
| **GM004413** | *odhA* | 2-oxoglutarate dehydrogenase | 1.6761 |  |
| **GM004445** | *dhbF* | diguanylate cyclase |  | 1.9629 |
| **GM004446** | *dhbB* | isochorismatase |  | 2.3013 |
| **GM004447** | *dhbE* | enterobactin synthase subunit E | 3.0117 |  |
| **GM004448** | *dhbC* | isochorismate synthase |  | 2.684 |
| **GM004449** | *dhbA* | 2, 3-dihydroxybenzoate-2, 3-dehydrogenase | 2.8893 |  |
| **GM004474** | *maeA2* | malate dehydrogenase |  | -1.3578 |
| **GM004689** | *phaA* | acetyl-CoA acetyltransferase | -2.431 |  |
| **GM004690** | *adc* | acetoacetate decarboxylase | -2.5533 |  |
| **GM004691** | *atoD* | branched-chain amino acid dehydrogenase | -2.3992 |  |
| **GM004692** | *ctfB* | acetate CoA-transferasc | -2.3947 |  |
| **GM004716** | *tuaD* | UDP-glucose6-dehydrogenase | 3.5632 | 4.576 |
| **GM004723** | *gmd* | GDP-D-mannose dehydratase | 2.191 | 3.9285 |
| **GM004724** | *ger2* | GDP-L-fucose synthase | 2.0923 | 3.9397 |
| **GM004732** | *manC* | mannose-1-phosphate guanylyltransferase | 2.0774 |  |
| **GM004744** | *gtaB* | UTP-glucose-1-phosphate uridylyltransferase | 2.6658 | 1.5664 |
| **GM005384** | *gpm* | phosphoglycerate kinase |  | 1.3145 |
| **GM005571** | *ldh3* | lactate dehydrogenase | -4.4277 | -3.6545 |
| **GM005578** | manZ | PTS mannose transporter subunit IID | -1.9322 |  |
| **GM005579** | *manY* | mannose permease IIC component | -2.2646 |  |
| **GM005580** | *manX* | phosphotransferase system enzyme Il, mannose-specific, factor IlAB | -2.5343 |  |
| **GM005581** | *manX* | phosphotransferase system, mannose/fructose/N-acetylgalactosamine-specific | -2.0365 |  |
