# Supplementary material for: Genome-wide mapping of GlnR-binding sites reveals the global regulatory role of GlnR in controlling the metabolism of nitrogen and carbon in Paenibacillus polymyxa WLY78
Source: BMC Genomics. 2023 Feb 23;24:85. doi: 10.1186/s12864-023-09147-1 (PMC9948412; doi:10.1186/s12864-023-09147-1)
Supplement: Supplementary file 3 — Additional file 3. The expression of genes in Paenibacillus polymyxa WLY78 in WT and delta glnR mutant under nitrogen excess and limited conditions. Table S1. Primers used in this study. Table S2. Transcription of genes involved in nitrogen fixation and metabolism. Table S3. Transcription of genes involved in carbon metabolism. Fig. S1. The original blots of EMSA results in Fig. 1B. Each lane contained 0.3 nM labeled probe. For the 5 lanes of the EMSA of each gene promoter, lanes 1 to 5 contained 0, 0.5, 1, 1, and 1 μM His6-GlnR respectively. A 200-fold excess of unlabelled specific probe (lanes 4) or nonspecific competitor DNA (Probe 1) (lanes 5) was used in competition assays. Fig. S2. The SDS-PAGE result of the purified His6-GlnR protein. The lanes from left to right are marker, solution eluted by lysis buffer containing 20 mM imidazole, solution eluted by 1 mL lysis buffer containing 250 mM imidazole from the first to the fourth time. Fig. S3. The titer of GlnR polyclonal antibody determined by ELISA. [file 12864_2023_9147_MOESM3_ESM.zip › Fig. S1.pptx]

## Slide 1
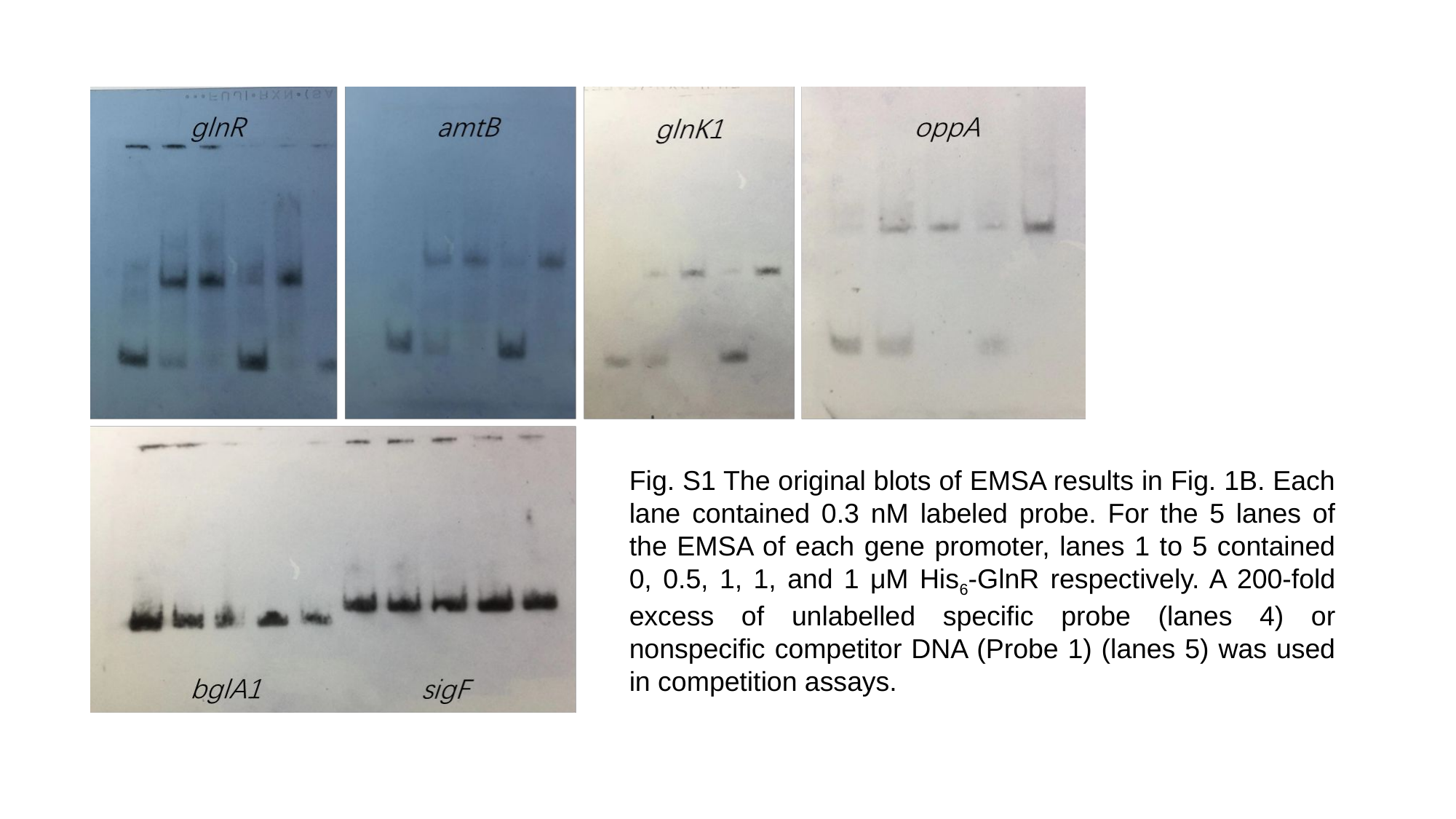

Fig. S1 The original blots of EMSA results in Fig. 1B. Each lane contained 0.3 nM labeled probe. For the 5 lanes of the EMSA of each gene promoter, lanes 1 to 5 contained 0, 0.5, 1, 1, and 1 μM His6-GlnR respectively. A 200-fold excess of unlabelled specific probe (lanes 4) or nonspecific competitor DNA (Probe 1) (lanes 5) was used in competition assays.
